# A Penalization Method for Estimating Heterogeneous Covariate Effects in Cancer Genomic Data

**DOI:** 10.3390/genes13040702

**Published:** 2022-04-15

**Authors:** Ziye Luo, Yuzhao Zhang, Yifan Sun

**Affiliations:** 1 School of Statistics, Renmin University of China, No. 59 Zhongguancun Street, Beijing 100872, China; 2017100369@ruc.edu.cn (Z.L.); 2016201698@ruc.edu.cn (Y.Z.); 2 Center for Applied Statistics, School of Statistics, Renmin University of China, No. 59 Zhongguancun Street, Beijing 100872, China

**Keywords:** heterogeneity, covariate effects, penalization, genomics

## Abstract

In high-throughput profiling studies, extensive efforts have been devoted to searching for the biomarkers associated with the development and progression of complex diseases. The heterogeneity of covariate effects associated with the outcomes across subjects has been noted in the literature. In this paper, we consider a scenario where the effects of covariates change smoothly across subjects, which are ordered by a known auxiliary variable. To this end, we develop a penalization-based approach, which applies a penalization technique to simultaneously select important covariates and estimate their unique effects on the outcome variables of each subject. We demonstrate that, under the appropriate conditions, our method shows selection and estimation consistency. Additional simulations demonstrate its superiority compared to several competing methods. Furthermore, applying the proposed approach to two The Cancer Genome Atlas datasets leads to better prediction performance and higher selection stability.

## 1. Introduction

The tremendous development of high-throughput sequencing techniques allows for the generation of massive genomic data, e.g., gene expressions and Single-Nucleotide Polymorphisms (SNPs). These data provide an unprecedented opportunity of uncovering biomarkers associated with outcomes such as the development and progression of complex diseases, e.g., cancers and type II diabetes. Numerous studies on this topic have been hitherto carried out. However, most existing studies assume that a covariate has an identical effect on the outcome variable for all subjects, which is often unrealistic in practice. For example, Ford et al. [1] found that the risk of breast and ovarian cancers in BRCA2 mutation carriers increases with age. Another example is that the effects of some genes in the nicotinic 15q25 locus on lung cancer risk are mediated by nicotine dependence [2]. These findings suggest that the effects of a specific covariate can be heterogenous and discrepancies in covariate effects or covariate-outcome associations may arise due to the differences in clinical characteristics and other traits that differ across subjects. As such, ignoring such effects, heterogeneity in genomic data analysis can result in biased estimations and misleading inferences.

The most commonly used strategy for handling heterogeneity is subgroup analysis, under which subjects form subgroups and each subgroup has unique covariate-outcome associations. A number of approaches have been proposed, such as the finite mixture model [3,4,5], and penalization-based approaches, such as concave fusion penalization [6,7], and C-Lasso [8]. However, these approaches assume that the effects of covariates are the same within each subgroup. As suggested by the literature, the covariate (e.g., genetic) effects are typically associated with clinical measures (e.g., age and number of cigarettes smoked per day), which are often continuous variables. As such, in some applications, covariate effects are more likely to vary smoothly rather than being locally constant within each subgroup.

In this study, we focus on a scenario where the subjects can be ordered by an auxiliary variable (see Section 2 for details). We consider a linear regression model with heterogeneous covariate effects by allowing the regression coefficients to vary smoothly across subjects. We then propose a novel penalization approach to capture the smoothing changes of coefficients. Under this approach, a “spline-lasso” penalty is imposed on the second-order derivatives of the coefficients to encourage smoothness in coefficients’ changes. Additionally, we introduce a penalty of the group Lasso form to accommodate the high dimensionality of genomic data (i.e., the number of genes is larger than the sample size) and select the relevant covariates.

**Figure 1:**
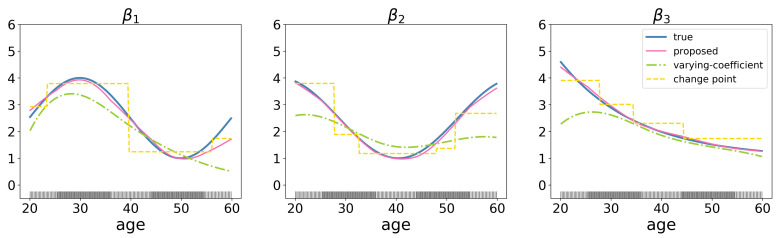
Estimation results for a toy example with N=200 subjects and p=10 genes with three important genes. The values of the gene expressions are generated from multivariate normal distribution N(0,Σ), where $\Sigma_{ii}=1$ and $\Sigma_{ij}=0.3^{\left|i-j\right|}$. The ticks on the *x*-axis represent the values of the auxiliary variable (age).

Our work is related to the varying coefficient models, a kind of classical semi-parametric model. It treats the coefficients as functions of certain characteristics, and uses various nonparametric smoothing techniques, such as spline-based methods [9,10], and local polynomial smoothing [11], to approximate the unknown coefficient functions. For example, high-dimensional varying coefficient models proposed by Wei et al. [12], Xue and Qu [13], Song et al. [14], Chen et al. [15], finite mixture of varying coefficient model [16], and additive varying-coefficient model for non linear gene-environment interactions [17]. Compared to these varying-coefficient regression approaches, the proposed method has few requirements for the distribution of auxiliary variables and better estimates the regression coefficients when auxiliary variable is unevenly distributed (Figure 1).

Moreover, the proposed approach is also related to but also significantly advances existing ones. First, it advances existing genomic marker identification studies by considering the heterogeneity of covariate effects. Second, it advances gene-environment interaction analysis methods [18,19] by allowing more flexibility in the relationship pattern (not limited to a given relationship) between covariate (genetic) effects and environmental factors (auxiliary variables). Finally, the proposed approach also advances the existing multiple changing-point regression studies [20,21] by tracking the gradually changes of coefficients rather than the abrupt ones (Figure 1). Overall, this approach is practically useful for analyzing genomic data and may lead to important new findings.

To further illustrate differences of the proposed method from varying-coefficient models and multiple changing-point regression methods, consider a simple simulation example with N=200,p=10, and 3 significant variables. The coefficient for each variable varies among individuals and is a function of a certain environmental factor, e.g., age. Suppose the age is unevenly distributed among subjects, with subjects concentrated between the age of 25–35 and 45–55, which is indicated by denser rugs in the Figure 1. We compare proposed method with the varying-coefficient model [12] and the change point regression model [22]. The simulation results show that the compared method performs relatively poorly (root mean squared errors (RMSE) = 4.853, rooted prediction error (RPE) = 1.325 for varying-coefficient model; RMSE = 3.158, RPE = 1.242 for change point regression model), while proposed method identifies the true coefficient pathway consistently (RMSE = 0.954, RPE = 0.893).

The rest of this paper is organized as follows. In Section 2, we introduce the proposed approach, present the algorithm, and discuss some theoretical properties. Simulations are shown in Section 3. Section 4 presents the analysis of two The Cancer Genome Atlas (TCGA) datasets. Section 5 concludes the paper. The technical details of proofs and additional numerical results are provided in the Appendix A, Appendix B, Appendix C and Appendix D.

## 2. Materials and Methods

Assume a dataset consists of *N* independent subjects. For subject *n*, let $y^{n}$ and $X^{n}=\left(X_{1}^{n},X_{2}^{n},\ldots ,X_{p}^{n}\right)$ denote the response variable and the *p*-dimensional vector of genomic measurements, respectively. In our numerical study, we analyze gene expression data. It is noted that the proposed approach can also be applied to other types of omics measurements. Assume the data has been standardized and consider a heterogenous linear regression model given by:

(1) $$y^{n}=X^{n}\beta^{n}+\varepsilon^{n},$$

where $\varepsilon^{n}$’s are independent and identically distributed (i.i.d.) random errors and $\beta^{n}={\left(\beta_{1}^{n},\beta_{2}^{n},\ldots ,\beta_{p}^{n}\right)}^{⊤}$ are the regression coefficients. Different from the standard regression model, which imposes an identical β on all subjects, model (1) allows $\beta^{n}$ to be subject-specific. Here, we consider a linear regression, which is standard to model the relationship between covariates and outcomes. The proposed approach is applicable to other models, for example, the AFT model. More details are provided in Appendix A. In this paper, we focus on a scenario where the heterogeneity analysis of covariate effects can be conducted with the aid of an auxiliary variable whose measurement is available for *N* subjects. Specifically, we assume that the subjects have been sorted according to the auxiliary variable’s values. Further, the effect of a relevant covariate on the response variable is expected to vary smoothly across subjects. The studies reviewed in Section 1 and other similar ones suggest that the covariate (e.g., genetic) effects are usually associated with clinical traits. As such, we choose an auxiliary variable with known interactions with clinical variables. Please see the examples in the data analysis section for details (Section 4).

**Remark** **1.** *In subgroup-level heterogeneity analysis, an auxiliary variable may not be needed. However, a subject-level heterogeneity analysis is intractable without the auxiliary variable due to non-identifiability. To date, the existing methods that can handle this type of heterogeneity, for example, varying-coefficients and interaction analysis, all require an auxiliary variable. Note that, in our analysis, the auxiliary variable does not need to be “precise.” Consider, for example, a sample of size 5. Auxiliary variable A has the values 1, 3, 7, 2, and 9 for the five subjects and auxiliary variable B has the values −0.8, 0.4, 0.5, 0.0, and 3. Although auxiliary variables A and B do not match, the proposed method can lead to the same covariate effects when using both auxiliary variables as an ordering index.*

As previously mentioned, we propose a novel penalized estimation and denote $\beta_{j}={\left(\beta_{j}^{1},\ldots ,\beta_{j}^{N}\right)}^{⊤}$ and $\beta ={\left(\beta_{1}^{⊤},\beta_{2}^{⊤},\ldots ,\beta_{p}^{⊤}\right)}^{⊤}$. Then, we define estimator $\hat{\beta }$ as the solution of the following optimization problem:

$$\begin{array}{ccc} \hat{\beta }=\arg\min_{\beta }F\left(\beta \right)\equiv & & \frac{1}{2N}\sum_{n=1}^{N}{\left(y^{n}-X^{n}\beta^{n}\right)}^{2}+\lambda_{1}\sum_{j=1}^{p}\omega_{j}{∥\beta_{j}∥}_{2}\\ & & +\lambda_{2}\sum_{j=1}^{p}\sum_{n=1}^{N}\frac{1}{2}{\left[\left(\beta_{j}^{n+1}-\beta_{j}^{n}\right)-\left(\beta_{j}^{n}-\beta_{j}^{n-1}\right)\right]}^{2}, \end{array}$$

where ${∥u∥}_{2}$ represents the two-norm of any vector *u* and $\omega_{j}$’s are weights. $\lambda_{1}\geq 0$ and $\lambda_{2}\geq 0$ are data-dependent tuning parameters. We also introduce an “expanded” measurement matrix Z:

$$Z={\left[\begin{array}{ccccccccc} X_{1}^{\left(1\right)} & \; & & X_{2}^{\left(1\right)} & \; & \cdots & X_{p}^{\left(1\right)}\\ \; & \ddots & \; & & \ddots & \; & & \ddots \\ \; & \; & X_{1}^{\left(N\right)} & \; & & X_{2}^{\left(N\right)} & \cdots & \; & X_{p}^{\left(N\right)} \end{array}\right]}_{N\times Np}.$$

We denote $Y={\left(y^{1},y^{2},\ldots ,y^{n}\right)}^{⊤}$. Then, objective function F(β) can be rewritten in a more compact form:

(2) $$F\left(\beta \right)=\frac{1}{2N}{∥Y-Z\beta ∥}_{2}^{2}+\lambda_{1}\sum_{j=1}^{p}\omega_{j}∥\beta_{j}{∥}_{2}+\frac{\lambda_{2}}{2}\sum_{j=1}^{p}{∥A\beta_{j}∥}_{2}^{2},$$

$A={\left\{e^{n}-2e^{n+1}+e^{n+2},n=1,2,\ldots ,N-2\right\}}^{⊤}$ with $e^{n}$ being the N×1 column vector, whose *n*th element is 1, and the others are 0.

Rationale. In (2), the first term is the lack-of-fit measure, expressed as the sum of *N* individual subjects. The first penalty is the group Lasso on β. Here the “group” refers to the regression coefficients of *N* subjects for a specific covariate. This penalty accommodates the high-dimensionality of the data and allows for the regularized estimation and selection of relevant covariates. The “all-in-all-out” property of the group Lasso leads to a homogeneous sparsity structure, that is, the *N* subjects have the same set of important covariates. To obtain an oracle estimator, we add weight $\omega_{j}$ to the sparsity penalty, which is determined by an initial estimator. Assuming that initial estimator $\tilde{\beta_{j}}$ is available, let $\omega_{j}=\frac{1}{∥{\tilde{\beta }}_{j}{∥}_{\infty }}$.

The main advancement is the second penalty, which has a spline form. It penalizes the second-order derivatives (in discrete version) of coefficients $\beta_{j}^{n}$ to promote the smoothness of coefficients between adjacent subjects. Note that the coefficients for any adjacent subjects are assigned a penalty of the same magnitude regardless of the distance between subjects measured by the auxiliary variable. Different from standard spline-lasso penalties [23], it is imposed on the regression coefficients of different subjects. Furthermore, different from some alternatives which promote first-order smoothness, such as the fusion Lasso [24] and smooth Lasso [25], this penalty encourages second-order smoothness. Additionally, the quadratic form of this penalty makes it computationally easier than the absolute-value-form penalty, such as Lasso. It is noted that the gene-environment interaction analysis also can capture the smooth change of covariate effects over an auxiliary variable (environmental factor). However, the interaction analysis approach requires specifying a parametric form of the relationship between covariate effects and auxiliary variable, which is not very flexible in practice, in particular, for high-dimensional data.

### 2.1. Computation

Optimization (2) can be realized using a block coordinate descent (CD) algorithm. For each covariate *j*, its measurement on the *N* subjects $X_{j}={\left(X_{j}^{1},X_{j}^{2},\ldots ,X_{j}^{N}\right)}^{⊤}$ forms a group and corresponding coefficients $\beta_{j}$ are simultaneously updated. The algorithm optimizes the objective function with respect to one group of coefficients and iteratively cycles through all groups until convergence is reached. Let $Z_{\left[j\right]}=\mathrm{diag}\left(X_{j}^{⊤}\right)$ represent the sub-matrix of Z, corresponding to $X_{j}$, which is a diagonal matrix. We denote $\beta_{j}^{\left(k\right)}$ as the estimate of $\beta_{j}$ in the *k*th iteration. The proposed algorithm proceeds as follows:

1. Initialize k=0, $\beta^{\left(k\right)}=0$ and set $\beta^{\left(-1\right)}=\beta^{\left(0\right)}$.
2. Update k=k+1. For j∈{1,2,…,p}, minimize $M(\beta_{j})$ with respect to $\beta_{j}$, where:
  $$\begin{array}{ccc} M(\beta_{j}) & = & L\left(\beta_{j}\right)+\lambda_{1}\omega_{j}{∥\beta_{j}∥}_{2},\\ L(\beta_{j}) & = & \frac{1}{2N}∥Y-Z_{\left[j\right]}\beta_{j}-\sum_{m>j}Z_{\left[m\right]}\beta_{m}^{\left(k-1\right)}-\sum_{m<j}Z_{\left[m\right]}\beta_{m}^{\left(k\right)}{∥}_{2}^{2}+\frac{\lambda_{2}}{2}{∥A\beta_{j}∥}_{2}^{2}. \end{array}$$
  This can be realized by executing the following steps:
  (a) Set the step size t=1.
    Compute
    $$\begin{array}{ccc} D_{1j} & = & \frac{1}{N}Z_{\left[j\right]}^{⊤}\left(\sum_{m\geq j}Z_{\left[m\right]}\beta_{m}^{\left(k-1\right)}+\sum_{m<j}Z_{\left[m\right]}\beta_{m}^{\left(k\right)}-Y\right)+\lambda_{2}A^{⊤}A\beta_{j}^{\left(k-1\right)},\\ G_{j} & = & {\left(1-\frac{t\lambda_{1}\omega_{j}}{∥\beta_{j}^{\left(k-1\right)}-tD_{1j}{∥}_{2}}\right)}_{+}\left(\beta_{j}^{\left(k-1\right)}-tD_{1j}\right). \end{array}$$
    Increase step size by t←0.8t until
    $$L\left(G_{j}\right)\leq L\left(\beta_{j}^{\left(k-1\right)}\right)+D_{1j}^{⊤}\left(G_{j}-\beta_{j}^{\left(k-1\right)}\right)+\frac{1}{2t}{∥G_{j}-\beta_{j}^{\left(k-1\right)}∥}_{2}^{2}.$$
  (b) Compute
    (3) $$\begin{array}{ccc} v & = & \beta_{j}^{\left(k-1\right)}+\frac{k-2}{k+1}\left(\beta_{j}^{\left(k-1\right)}-\beta_{j}^{\left(k-2\right)}\right),\\ D_{2j} & = & \frac{1}{N}Z_{\left[j\right]}^{⊤}\left(Z_{\left[j\right]}v+\sum_{m>j}Z_{\left[m\right]}\beta_{m}^{\left(k-1\right)}+\sum_{m<j}Z_{\left[m\right]}\beta_{m}^{\left(k\right)}-Y\right)+\lambda_{2}A^{⊤}Av \end{array}$$
    and update the estimate of $\beta_{j}$ by
    $$\beta_{j}^{\left(k\right)}\leftarrow {\left(1-\frac{t\lambda_{1}\omega_{j}}{∥v-tD_{2j}{∥}_{2}}\right)}_{+}\left(v-tD_{2j}\right).$$
3. Repeat Step 2 until convergence is achieved. In our numerical study, the convergence criterion is $\min_{1\leq j\leq p}{∥\beta_{j}^{\left(k\right)}-\beta_{j}^{\left(k-1\right)}∥}_{2}<10^{-3}$.

To speed up the algorithm, we add a momentum term to the last iteration of $\beta_{j}^{\left(k-1\right)}$ in (3) and determine step size *t* via the backtracking line search method. After the algorithm converges, some groups of coefficients are estimated as zeros. To further improve estimation accuracy, in practice, we can remove the covariates with zero coefficients and re-estimate the nonzero coefficients by minimizing objective function (2) without the sparsity penalty. The proposed approach involves two tuning parameters selected using a grid search and the *K*-fold cross validation with K=5.

**Figure A1:**
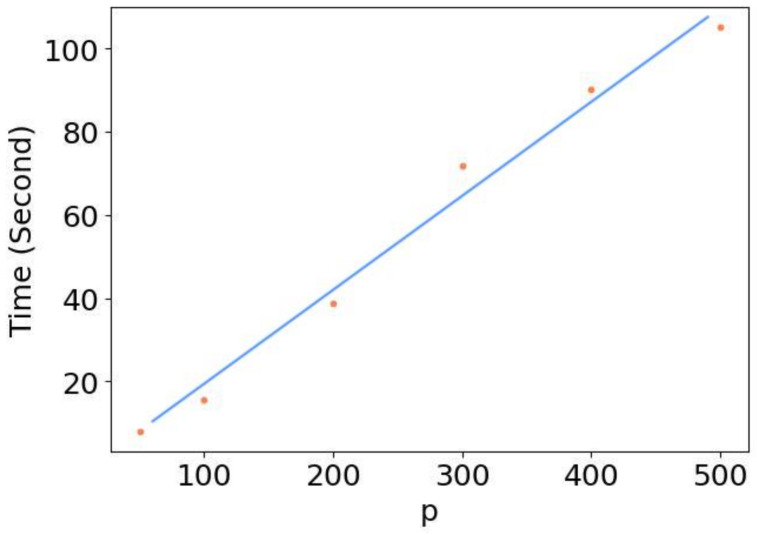
Simulation results: computation time of the proposed approach as a function of the number of features *p* for five replicates under Scenario 6 with N=200,q=20,ρ=0.3. The red dots represents the computation time under corresponding variable dimension, and the blue line represents the fitted value.

Realization. To facilitate data analysis within and beyond this study, we have developed a Python code implementing the proposed approach and made it publicly available at https://github.com/foliag/SSA (accessed on 21 March 2022). The proposed approach is computationally affordable. As shown in Figure A1, the computational time of the proposed approach is linear, with an increasing number of features.

### 2.2. Statistical Properties

Here, we establish the consistency properties of the proposed approach. We define a new dataset $\left(\tilde{Y},\tilde{Z}\right)$ by ${\tilde{Y}}_{\left(n+(n-2)\times p\right)}={\left(Y,0\right)}^{⊤}$ and ${\tilde{Z}}_{(n+(n-2)\times p)\times np}={\left(Z,\sqrt{N\lambda_{2}}A\right)}^{⊤}$, where $A=A⊗I_{p\times p}$. Then, objective function (2) can be converted to an adaptive group Lasso form:

$$F\left(\beta \right)=\frac{1}{2N}∥\tilde{Y}-\tilde{Z}{\beta ∥}_{2}^{2}+\lambda_{1}\sum_{j=1}^{p}\omega_{j}{∥\beta_{j}∥}_{2}.$$

Let $\beta^{0}={\left({\left(\beta_{1}^{0}\right)}^{⊤},{\left(\beta_{2}^{0}\right)}^{⊤},\ldots ,{\left(\beta_{p}^{0}\right)}^{⊤}\right)}^{⊤}$ be the true parameter values. We denote *q* as the number of non-zero coefficient vectors. Without loss of generality, assume $\beta_{j}^{0}\neq 0$ for 1≤j≤q. We define two sets, $E_{1}=\left\{j|1\leq j\leq q\right\}$ and $E_{0}=\left\{j|q+1\leq j\leq p\right\}$, corresponding to the index of nonzero and zero coefficient vectors, respectively. Let $J=A^{\prime }A$ and $\Sigma =\frac{1}{N}Z^{⊤}Z+\lambda_{2}J$. We then use τ to represent the minimal eigenvalue of matrix Σ. The following conditions are assumed:

(C0) Errors $\varepsilon^{1},\varepsilon^{2},\ldots ,\varepsilon^{N}$ are i.i.d sub-Gaussian random variables with mean zero. That is, for certain constants 0.5≤t≤1 and K,C≥0, the tail probabilities of $\varepsilon^{n}$ satisfy $P(|\varepsilon^{n}\left|>x\right)\leq Ke^{-Cx^{t}}$ for all x≥0 and n=1,2,…,N.
(C1) Let $m=\max_{1\leq j\leq p,1\leq n\leq N}\left|X_{j}^{n}\right|$. Then, m=O(1).
(C2) Let $\alpha_{1}=\min_{j\in E_{1}}\frac{∥\beta_{j}^{0}{∥}_{2}}{\sqrt{N}}$. Then, $\alpha_{1}=O\left(1\right)$. Moreover, there exists a constant $\alpha_{2}>0$ so that $P(\min_{j\in E_{1}}∥{\tilde{\beta }}_{j}{∥}_{\infty }>\alpha_{2})\to 1$.
(C3) τ>0 and $\frac{\lambda_{2}}{\tau }\to 0$.
(C4) $∥J\beta^{0}{∥}_{2}=O\left(\sqrt{N}\right)$.

Condition (C0) is the sub-Gaussian condition is commonly assumed in studies [26]. Condition (C1) assumes the measurement matrix is bounded. Similar conditions have been considered by AuthMartinussen and Scheike [27] and Binkiewicz and Vogelstein [28]. Condition (C2) puts a lower bound on the size of the smallest signal and assumes the initial ${\tilde{\beta }}_{j}$ is not too small for $j\in E_{1}$. Similar conditions have been considered by Wei and Huang [29]. Condition (C3) is similar to the assumption made in Case I of Guo et al. [23], which requires Σ to be invertible and the minimal eigenvalue τ to converge to 0 at a rate controlled by $\lambda_{2}$. Condition (C4) makes a weak constraint on $\beta^{0}$, which can be satisfied when for any nonzero coefficient vector $\beta_{k}$ ($k\in E_{1}$) the largest gap between two adjacent components is bounded.

**Theorem** **1.** *Assume Conditions (C0)–(C4) hold, as does event $\Omega =\left\{\max_{j\in (1,2,\ldots ,p)}{∥{\tilde{\beta }}_{j}∥}_{\infty }=o\left(\frac{N^{\frac{3}{4}}\lambda_{1}}{\log N\sqrt{\log p}}\right)\right\}$ when N does to infinity. We define $∥\beta^{0}-\hat{\beta }{∥}_{2,N}=\frac{∥\beta^{0}-\hat{\beta }{∥}_{2}}{\sqrt{N}}.$ Then, with a probability converging to one, we have*

$$∥\beta^{0}-\hat{\beta }{∥}_{2,N}\leq \frac{4\lambda_{1}\sqrt{q}\alpha_{2}^{-1}+2\lambda_{2}{∥J\beta^{0}∥}_{2}}{\tau \sqrt{N}}.$$

The proof is provided in Appendix B. If *q* is not too large and $\alpha_{2}$ and τ are not too small, we may have $\frac{\sqrt{q}}{\tau \alpha_{2}}$∼$o\left(\frac{N^{\frac{5}{4}}}{\log N\sqrt{\log p}}\right)$ (more details below). Then, we can find a $\lambda_{1}$ that satisfies $\frac{1}{\lambda_{1}}$∼$o\left(\frac{N^{\frac{3}{4}}}{\log N\sqrt{\log p}}\right)$ and $\lambda_{1}$∼$o\left(\tau \alpha_{2}\sqrt{\frac{N}{q}}\right)$ simultaneously. It is not difficult to prove that event Ω holds for the marginal regression estimator as the initial estimator. As a result, under conditions (C3) and (C4), the gap between $\beta^{0}$ and $\hat{\beta }$ converges to 0. This theorem thus establishes estimation consistency.

The following additional conditions are assumed:

(C5) Initial estimators ${\tilde{\beta }}_{j}$ are *r*-consistent for the estimation of certain $\xi_{j}$:
  $$r\max_{j\in E_{0}}{∥{\tilde{\beta }}_{j}-\xi_{j}∥}_{\infty }=O_{p}\left(1\right),\;\;r\to \infty ,$$
  where $\xi_{j}$ is an unknown constant vector satisfying $\max_{j\in E_{0}}{∥\xi_{j}∥}_{\infty }\leq M$.
(C6) Constants $\left\{p,q,M,\lambda_{1},\lambda_{2},\tau ,\alpha_{2}\right\}$ satisfy:
  $$\frac{\sqrt{q}\log N}{\tau N^{\frac{5}{4}}}+\frac{\lambda_{1}}{\tau \alpha_{2}}\sqrt{\frac{q}{N}}+\frac{\log N\sqrt{\log(p-q)}\left(N+q\tau^{-1}\right)}{N^{\frac{9}{4}}\lambda_{1}}\left(\frac{1}{r}+M\right)\to 0,$$
  $$\frac{2m^{2}\sqrt{q}(\lambda_{1}\alpha_{2}^{-1}\sqrt{q}+\lambda_{2}∥J\beta^{0}{∥}_{2})}{\tau \lambda_{1}\sqrt{N^{3}}}\left(\frac{1}{r}+M\right)\leq 1.$$

Condition (C5) is similar to condition (A2) in Huang et al. [26], which ensured that weight $\omega_{j}\approx \frac{1}{∥\xi_{j}{∥}_{\infty }}$ is not too small for $j\in E_{0}$. Condition (C6) restricts the numbers of covariates with zero and nonzero coefficients, the penalty parameters, the minimal eigenvalue of Σ, and the smallest nonzero coefficient. Given all conditions in Theorems 1 and 2, we may assume $\lambda_{1}=O\left(N^{-a}\right)$, $\lambda_{2}=O\left(N^{-b}\right)$, and $\tau =O(N^{c})$ for some 0<c<b<a<0.5; then, the number of nonzero coefficients *q* can be as large as $N^{d}$ for some $0\leq d\leq \frac{2(1-a+b-c)}{3}$. In this case, there can be $O(e^{N^{\frac{1}{2}-\delta }})$ zero coefficients, where δ is a small nonzero constant, assuming $\alpha_{2}=O\left(N^{\frac{d-1}{2}}\right)$ and M=O(1).

**Theorem** **2.** *Under Conditions (C0)–(C6),*

$$P\left(∥{\hat{\beta }}_{j}{∥}_{2}\neq 0,j\in E_{1},{∥{\hat{\beta }}_{j}∥}_{2}=0,j\in E_{0}\right)\to 1.$$

The proof is provided in Appendix C. This theorem establishes the selection consistency properties of the proposed approach under a high-dimensional setting.

## 3. Simulation

We set p=500. The data are generated from the following true model:

(4) $$y^{n}=\sum_{j=1}^{q}X_{j}^{n}\beta_{j}^{n}+\varepsilon^{n},\;n=1,2,\ldots ,N,$$

where the random errors are simulated independently from N(0,1). We investigate nine scenarios for the coefficients as follows:

Scenario 1. The coefficients are generated from trigonometric functions; for n=1,2,…,N,
  $$\beta_{j}^{n}=\left\{\begin{array}{cc} 1.5\sin(\frac{20\pi }{N}u_{j}^{n})+2.5 & j=1,\ldots ,\frac{q}{4}\\ 1.5\cos(\frac{17\pi }{N}u_{j}^{n}+0.4)+2.5 & j=\frac{q}{4}+1,\ldots ,\frac{q}{2}\\ 1.5\sin(\frac{17\pi }{N}u_{j}^{n}-1.2)+2.5 & j=\frac{q}{2}+1,\ldots ,\frac{3q}{4}\\ 1.5\cos(\frac{20\pi }{N}u_{j}^{n}-2)+2.5 & j=\frac{3q}{4}+1,\ldots ,q, \end{array}\right.$$
  where $u_{j}^{n}=a_{j}+\frac{N}{10}\cdot n,\;a_{j}$∼U(0,0.5).
Scenario 2. The coefficients are generated from exponential functions:
  $$\beta_{j}^{n}=\left\{\begin{array}{cc} 4\exp(-u_{j}^{n})+1 & j=1,\ldots ,\frac{q}{4}\\ 4\exp(-0.9u_{j}^{n})+1 & j=\frac{q}{4}+1,\ldots ,\frac{q}{2}\\ 4\exp(-0.8u_{j}^{n})+1 & j=\frac{q}{2}+1,\ldots ,\frac{3q}{4}\\ 4\exp(-0.7u_{j}^{n})+1 & j=\frac{3q}{4}+1,\ldots ,q, \end{array}\right.$$
  where $u_{j}^{n}=a_{j}+\frac{N}{100}\cdot n,\;a_{j}$∼U(0,0.2).
Scenario 3. The coefficients are generated from logarithmic functions:
  $$\beta_{j}^{n}=\left\{\begin{array}{cc} 0.5\ln{\left(u_{j}^{n}\right)}^{3}+1 & j=1,\ldots ,\frac{q}{4}\\ 0.5\ln{\left(u_{j}^{n}\right)}^{2.9}+1 & j=\frac{q}{4}+1,\ldots ,\frac{q}{2}\\ 0.5\ln{\left(u_{j}^{n}\right)}^{2.7}+1 & j=\frac{q}{2}+1,\ldots ,\frac{3q}{4}\\ 0.5\ln{\left(u_{j}^{n}\right)}^{2.5}+1 & j=\frac{3q}{4}+1,\ldots ,q, \end{array}\right.$$
  where $u_{j}^{n}=a_{j}+\frac{N}{20}\cdot n,\;a_{j}$∼U(0.7,0.9).
Scenario 4. The coefficients are generated from linear functions:
  $$\beta_{j}^{n}=\left\{\begin{array}{cc} 0.16u_{j}^{n}+2 & j=1,\ldots ,\frac{q}{4}\\ 0.15u_{j}^{n}+2 & j=\frac{q}{4}+1,\ldots ,\frac{q}{2}\\ 0.14u_{j}^{n}+2 & j=\frac{q}{2}+1,\ldots ,\frac{3q}{4}\\ 0.13u_{j}^{n}+2 & j=\frac{3q}{4}+1,\ldots ,q, \end{array}\right.$$
  where $u_{j}^{n}=a_{j}+\frac{N}{10}\cdot n,\;a_{j}$∼U(0,1).
Scenario 5. The coefficients are constants:
  $$\beta_{j}^{n}=\left\{\begin{array}{cc} 3a_{j}+2 & j=1,\ldots ,\frac{q}{2}\\ 2a_{j}+2 & j=\frac{q}{2}+1,\ldots ,q, \end{array}\right.$$
  where $a_{j}$∼U(0,1).
Scenario 6. The coefficients are generated from the four above (trigonometric, exponential, logarithmic and linear) functions, respectively. Each function generates an equal number of coefficients.
Scenario 7. The coefficients are generated from the four above functions, where 40% and 35% of the coefficients are generated from the trigonometric and linear functions, respectively, and 10% and 15% of the coefficients are generated from the exponential and logarithmic functions, respectively.
Scenario 8. The coefficients are generated from the four functions. The trigonometric, exponential, logarithmic, and linear functions generate 35%, 15%, 20%, and 30% of the coefficients, respectively.
Scenario 9. The coefficients are generated as in Scenario 5. We select 40% of the coefficients and, for each function, add random perturbations on their values in one or two ranges, where each range includes 20 consecutive subjects.

**Figure A2:**
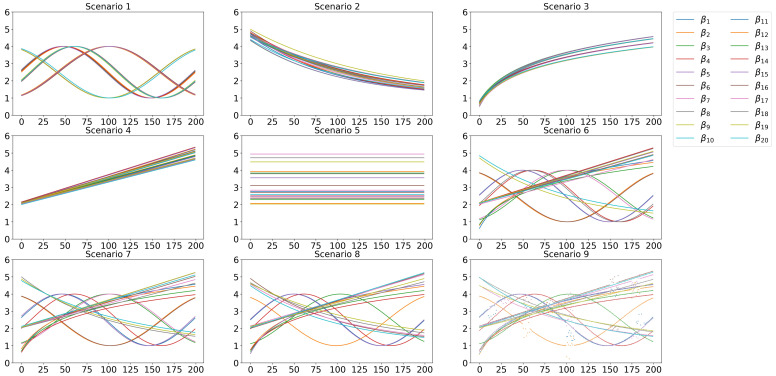
Nonzero coefficients of all subjects under Scenarios 1–9. The *x*-axis represents the subjects, and the *y*-axis represents the coefficient values.

In Scenarios 1–5, the *q* coefficients are generated from the same function, whereas from different functions in Scenarios 6–9. The coefficients in Scenario 5 are constants, that is, there is no heterogeneity in covariate effects. Some of coefficients in Scenario 9 do not change smoothly across subjects, but have a few discontinuous areas. Figure A2 presents q=20 nonzero coefficients as a function of N=200 subjects under nine scenarios. In the first eight scenarios, the *p* covariates are generated from a multivariate normal distribution with marginal mean 0 and variance 1. We consider an auto-regressive correlation structure, where covariates *j* and *k* have the correlation coefficient $\rho^{\left|j-k\right|}$ with ρ=0.3 and 0.8, corresponding to the weak and strong correlations, respectively. In Scenario 9, the *p* covariates are generated independently from a uniform distribution on (−1,1). It is noted that the aforementioned nonlinear functions of regression coefficients are widely used in simulation studies of varying-coefficient models for genomic data [30,31].

We consider two versions of the proposed approach. One uses the “standard” Lasso to obtain the initial estimator of coefficients (New-Lasso) and the other uses marginal regression (New-Mar). Both estimators are homogeneous, that is, the coefficients are the same for all subjects. To better gauge the proposed approach, we compare it with three alternatives: (a) Lasso, which directly applies the Lasso method to the entire dataset but does not account for the heterogeneity of coefficients across different subjects; (b) AdLasso, which is the group adaptive Lasso in the varying-coefficient model [12]; and (c) IVIS, which uses the independent screening technique for fitting the varying-coefficient model [14]. The last two methods focus on variable selection and the estimation of the varying-coefficient model in high-dimensional settings, where each nonzero coefficient is assumed a smooth function of a known auxiliary variable.

For the proposed approach and its alternatives, we evaluate the variable selection performance by TP (number of true positives) and FP (number of false positives). Estimation and prediction are also evaluated. Specifically, estimation is measured by RMSE (root mean squared errors), defined as $\sqrt{\frac{1}{p}\sum_{j=1}^{p}{∥\beta_{j}-{\hat{\beta }}_{j}∥}^{2}}$, and prediction is measured by RPE (root prediction errors), defined as $\sqrt{\frac{1}{N}\sum_{n=1}^{N}{\left(y^{n}-X^{n}{\hat{\beta }}^{n}\right)}^{2}}$.

Table 1 summarizes the simulation results over 100 replications for settings with N=200, q=20, and ρ=0.3. The rest of the results are presented in Table A1, Table A2 and Table A3. Across the simulation spectrum, the proposed approach has superior performance in terms of variable selection, as it can identify more important variables while having a low number of false positives. For example, in Scenario 1, N=200 and ρ=0.3 (Table 1), New-Lasso has (TP, FP) = (18.44, 0.16), while Lasso has (TP, FP) = (14.56, 0.30), AdLasso (TP, FP) = (16.64,0.70), and IVIS (TP, FP) = (13.76, 3.28). Consider another example, Scenario 9, N=200 and q=20 (Table 1). For the identification of important variables, the four approaches have the TP values 18.30 (New-Lasso), 15.40 (Lasso), 15.74 (AdLasso), and 14.24 (IVIS), and FP values 0.00 (New-Lasso), 2.60 (Lasso), 0.40 (AdLasso), and 4.64 (IVIS), suggesting the proposed approach is robust to perturbations. In most scenarios, New-Lasso outperforms New-Mar when covariates are weakly correlated (ρ=0.3), but performs worse than New-Mar when covariates are strongly correlated (ρ=0.8). These results stem from the fact that Lasso is not good at dealing with highly correlated covariates. In practice, we can select one of them according to the correlations among covariates. Examples are provided in Section 4. Lasso identifies a reasonable number of important variables but with higher false positive than the proposed approach. AdLasso shows a good performance in variable selection, but inferior to that of the proposed approach under most simulation settings. IVIS has the worst performance among the five approaches.

**Figure A3:**
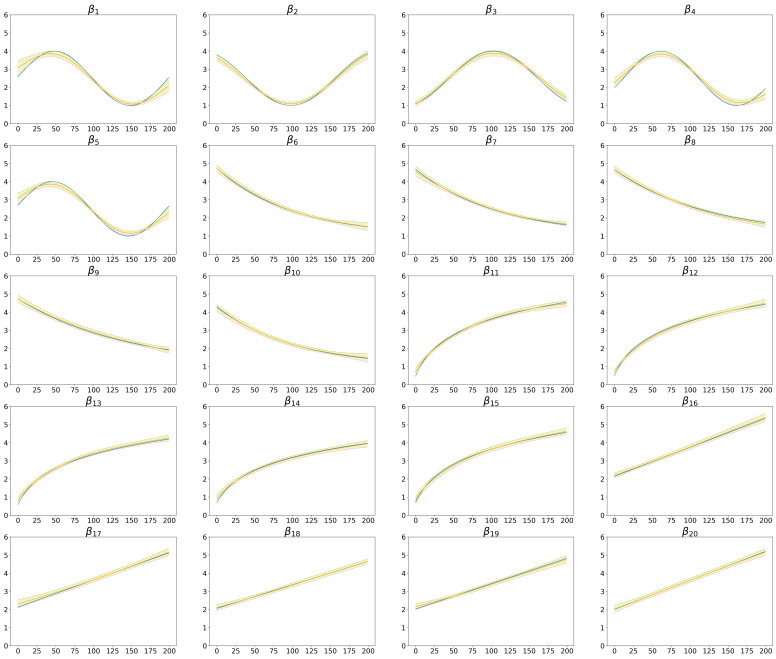
Estimated coefficients under Scenario 5 with N=200, q=20, and ρ = 0.3. The blue lines represent the true coefficients, the orange ones the coefficients estimated by New-Lasso, and the shadowed areas the 95% confidence intervals. The *x*-axis represents the subjects, and the *y*-axis represents the coefficient values.

**Figure A4:**
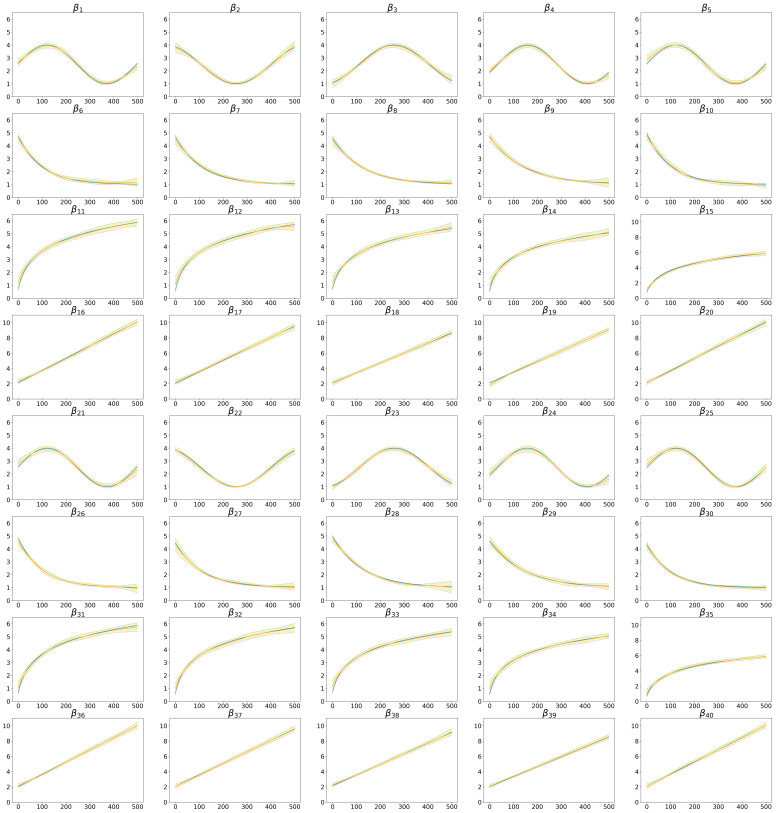
Estimated coefficients under Scenario 5 with N=500, q=40, and ρ = 0.8. The blue lines represent the true coefficients, the orange ones the coefficients estimated by New-Mar, and the shadowed areas the 95% confidence intervals. The *x*-axis represents the subjects, and the *y*-axis represents the coefficient values.

In the evaluation of estimation, the proposed approach again has a favorable performance. We plot the estimated nonzero coefficients as a function of subjects and 95% point-wise confidence intervals (Figure A3). In Scenario 6 with N=200, q=20, and ρ=0.3, the estimated coefficients are close to the true ones, and the confidence intervals contain the true coefficients for most subjects. However, the estimation results become worse for the coefficients of the first and last few subjects. This is because the information available to estimate these coefficients is less than that on the intermediate coefficients. This problem can be alleviated by increasing the sample size (Figure A4). Additionally, the proposed approach outperforms the alternatives in terms of prediction under most scenarios.

Overall, simulation suggests favorable performance of the proposed approach. It is interesting to note that it has satisfactory performance even under the no heterogeneity scenario (Scenario 5). Thus, it provides a safe choice for practical data analysis where the degree of heterogeneity in covariate effects is unknown. The other simulation settings have similar results. However, due to space constraints, we do not describe them here.

## 4. Data Analysis

Here, we apply the proposed approach to two TCGA datasets. As a cancer genomics program initiated by the National Institute of Health (NIH), TCGA publishes high quality clinical and genetic data. In our analysis, the data are downloaded from the cBioPortal website (http://www.cbioportal.org/, accessed on 16 January 2021) via the cgdrs package.

### 4.1. SKCM Data

Cutaneous melanoma (SKCM) is a cancer of the skin cells called melanocytes, leading to the majority of deaths from skin cancers. In our analysis, we are interested in the regulation of Breslow thickness, a measure of the size of melanoma growth, by gene expressions. We use age as the auxiliary variable, which is correlated with the melanoma development and progression [32]. After removing missing values from the Breslow thickness and age, a total of 228 patients are included in analysis. The median age is 58 (range: 18–90 years) and the median Breslow thickness is 2.45 (range: 0.28–75). All patients are sorted by age in ascending order. There are some patients that have the same age, but there are only a few (2–8) patients with the same age. The analysis results show that the orders of patients within each age have little impact on the identification of important genes and the effect estimation. Consequently, in the analysis, we sort the patients with the same age randomly. A total of 20,531 RNAseq gene expression measurements are available. More specifically, the processed level-3 gene expression data is used. Please refer to literature [33] for detailed information on generation and processing of gene expression data. To improve the reliability of the results, we conduct a marginal screening to screen out irrelevant genes and include 400 genes with lowest *p*-values in the downstream analysis. The gene expressions are assumed to connect with the response variable via a linear model.

**Figure 2:**
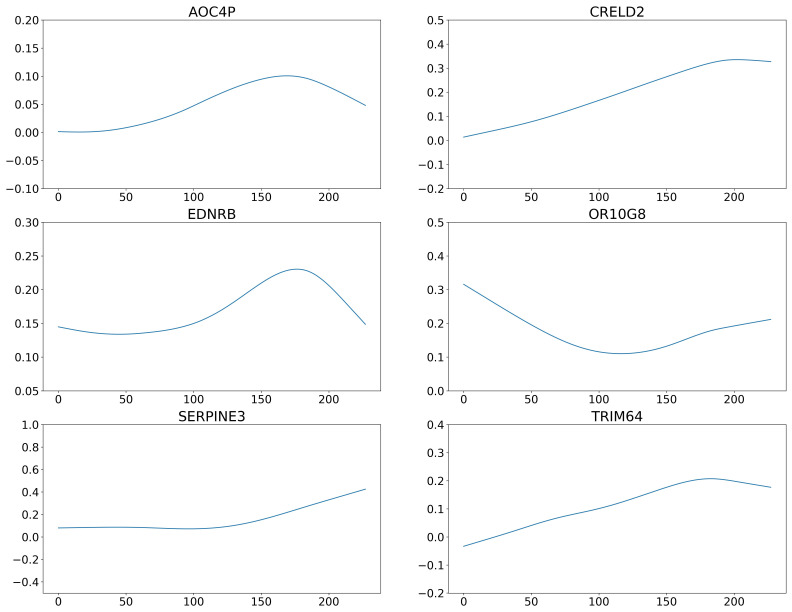
Analysis of the SKCM data using the proposed approach: estimated coefficients of the 6 genes for all subjects. The *x*-axis represents the subjects, and the *y*-axis represents the coefficient values.

The average correlation coefficient of 400 genes is 0.07, which is close to the 0.06 from the above simulation studies with ρ=0.3. As such, we adopt the New-Lasso method, which identifies 6 important genes. Figure 2 shows the estimated coefficients for the 6 genes. The changes in the effects of genes across patients are prominent, which suggests that the heterogenous model is more appropriate for this dataset. We observe different change patterns for the effects of the 6 genes. Specifically, genes AOC4P and EDNRB first increase then decrease; genes CRELD2 and TRIM64 show an increase then remain steady, while gene SERPINA3 demonstrate the opposite pattern, and effect of gene OR10GB has a bowl-shaped pattern. The literature suggests that the identified genes are biologically meaningful. For example, gene EDNRB provides instructions for making a protein called endothelia receptor type B. Inherited variations in this gene may be associated with an increased risk of melanomas [34]. Recent studies revealed that gene AOC4P plays critical roles at multiple levels in diverse physiological and pathological processes [35]. Some of changes in metastatic melanomas were identified in gene SERPINA3 encoding proteins involved in the regulation of the extracellular matrix [36]. A high SERPINA3 expression correlates with shorter disease survival [37,38], suggesting the SERPINA3 expression can be used as a prognostic marker in melanoma.

We also apply the alternatives described above. The comparative results are provided in Table A4. The different methods identify different sets of genes. Based on real data, the true set of important genes is unknown and, thus, it is difficult to directly evaluate the identification and estimation accuracy. To verify the results, we now evaluate prediction and stability. Specifically, the dataset is split into a training set and a testing set of sizes 2:1. The regression coefficients are estimated using the training set and used to make predictions for the subjects in the testing set. We repeat the process 50 times and calculate the average root prediction errors (RPEs) to be 0.775 (New-Lasso), 1.072 (Lasso), 1.036 (AdLasso), and 1.393 (IVIS). The proposed approach has the best prediction performance. Moreover, for the proposed approach, we compare the RPEs of training sets and that of testing sets, and no significant differences are found (*p*-value > 0.5), suggesting that the proposed approach does not produce obvious over-fitting. Additionally, we compute the observed occurrence index (OOI) values to evaluate the stability of the identification results. Figure A6 shows the OOIs of all methods. The proposed approach significantly outperforms the alternatives in terms of identification stability.

### 4.2. LUAD Data

Lung adenocarcinoma (LUAD) is a form of non-small cell lung cancer, being the most common type of lung cancer. In our analysis, survival time is the response variable. There are a total of 231 patients, sorted by their forced expiratory volume in one second (FEV1), an important measure of lung function. The median follow-up time is 20 (range: 0.13–232 months) and the median FEV1 is 83 (range: 1.95–156). A total of 18, 325 RNAseq gene expressions are initially available for the analysis. Using the same marginal screening process as described above, the number of gene expressions is reduced to 400.

**Figure A5:**
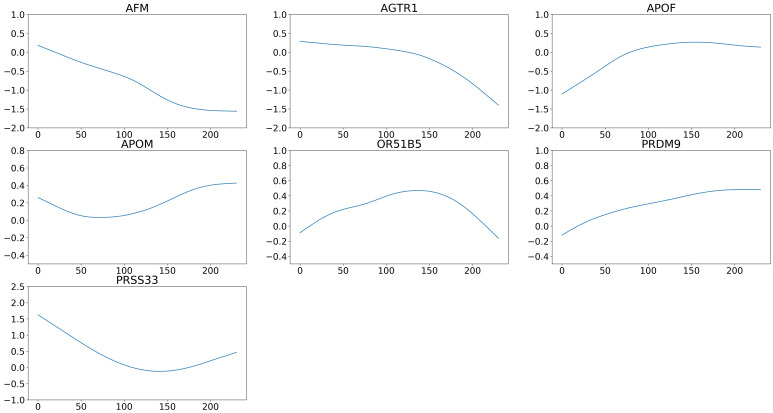
Analysis of LUAD data using the proposed approach: estimated coefficients of the 7 genes for all subjects. The *x*-axis represents the subjects, and the *y*-axis represents the coefficient values.

We adopt the accelerated failure time (AFT) model for the analysis of these censored survival data. The estimation procedure described above can be directly applied to the AFT model (see Appendix C). Because the genes have an average correlation coefficient (0.16) higher than that in the simulation studies with ρ=0.8 (≈0.13), the New-Mar method is used here. The proposed method identifies 7 genes. The estimated coefficients of the 7 genes are presented in Figure A5.

Extant studies provide biological evidence for the association of identified genes with lung cancer. For example, AGTR1, the gene encoding angiotensin II receptor type I, has been extensively studied in human cancers [39] and has shown a strong influence on tumor growth, angiogenesis, inflammation and immunity [40]. Guo et al. [41] shows that methylation profiles of AGTR1 could be an effective methylation-based assay for non-small cell lung cancer diagnosis.

**Figure A6:**
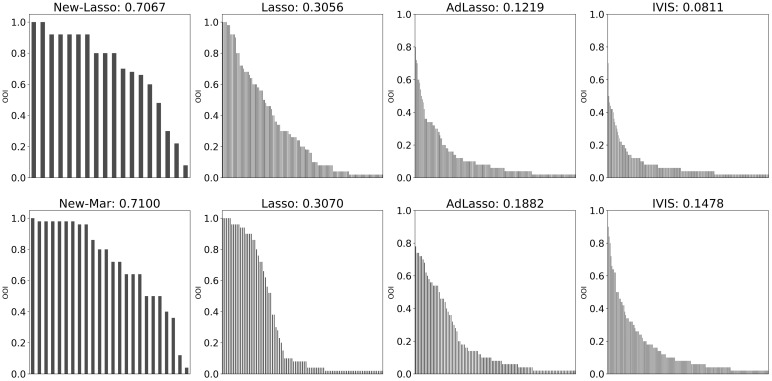
OOIs in the data analysis. Top: SKCM, bottom: LUAD.

Data are also analyzed using the alternative methods. The summary comparison results (Table A4) again suggest that different methods produce different results. With censored survival data, we use the log-rank statistics to measure prediction performance. The higher log-rank statistics indicate better prediction performance and the proposed approach has an average log-rank statistic of 11.67, compared with 4.43 for Lasso, 5.81 for AdLasso and 3.08 for IVIS. The OOI results are also presented in Figure A6. The proposed approach has again the highest OOI among all methods.

### 4.3. Simulation on SKCM Dataset

It has been recognized in some studies that simulated data may be “simpler” than real data. Here, we conduct an additional set of simulation based on the SKCM data analyzed above. Specifically, the observed gene expression data and the estimated coefficients in Section 4.1 are used in simulation. The simulation results are summarized in Table A5. It is observed that the proposed method maintains a relative edge over the alternatives, which justifies the effectiveness of the proposed method.

## 5. Discussion

The mature application of the high-throughput technology has produced a large amount of genomic data. With the rapid development of precision medicine, the heterogeneity effect of covariates has received increasing attention in disease genomic studies. However, most existing studies focus on the subgroup-specific effects, meaning the effects are the same within each subgroup, thus neglecting the possible varying effects within a subgroup. In this paper, we consider that the effects of covariates change smoothly across subjects. We thus propose a novel penalization-based estimation method, which combines a group-lasso penalty and a spline-lasso penalty based on subgroup-based studies by capturing the varying effects within each subgroup. It also advances the existing varying-coefficient studies by lowering the requirements for the distribution of the auxiliary variable. We show that, under the appropriate conditions, the proposed approach can correctly select important covariates with a probability converging to one and estimates the coefficients consistently. Simulations demonstrated a satisfactory practical performance and data analysis led to sensible findings, significantly different from those using alternative methods.

WIth the proposed regression model, it is impossible to estimate directly the subject-specific covariate effects due to the non-identifiability problem. This is resolved by introducing an auxiliary variable, which can have a biological interpretation. As such, it would be of interest to develop other frameworks that can differentiate between heterogeneous covariate effects in the (partial) absence of auxiliary variable. Additionally, the data analysis results also warrant further investigation.

## Figures and Tables

**Table 1:** Simulation results for *N* = 200, *p* = 500, q=20, and ρ=0.3. Each cell shows the mean (sd). The bold represents the best value.

Scenario	Method	TP	FP	RMSE	RPE
1	Lasso	14.57 (1.39)	0.30 (0.67)	6.56 (0.69)	12.92 (1.80)
	AdLasso	16.64 (1.22)	0.71 (0.95)	4.69 (0.34)	8.41 (0.47)
	IVIS	13.76 (1.31)	3.29 (0.66)	5.91 (0.74)	11.17 (0.84)
	New-Lasso	**18.45 (1.36)**	**0.17 (0.03)**	**2.34 (0.21)**	**1.92 (0.29)**
	New-Mar	16.14 (2.16)	1.84 (0.53)	3.98 (0.43)	3.52 (0.38)
2	Lasso	14.43 (1.45)	**0.00 (0.00)**	6.30 (0.86)	12.38 (2.12)
	AdLasso	17.50 (0.86)	0.69 (0.84)	4.74 (0.48)	8.54 (0.62)
	IVIS	14.20 (0.92)	3.10 (0.88)	5.85 (0.66)	10.23 (0.90)
	New-Lasso	**19.76 (0.44)**	**0.00 (0.00)**	**0.98 (0.20)**	**1.02 (0.32)**
	New-Mar	18.03 (1.88)	2.40 (0.42)	2.82 (0.53)	2.34 (0.40)
3	Lasso	14.35 (1.76)	0.15 (0.37)	7.24 (0.90)	13.70 (2.42)
	AdLasso	16.90 (1.27)	0.30 (0.53)	5.44 (0.66)	9.64 (0.91)
	IVIS	14.99 (0.89)	3.58 (0.91)	6.32 (0.71)	11.37 (0.96)
	New-Lasso	**19.81 (0.41)**	**0.00 (0.00)**	**1.02 (0.21)**	**1.02 (0.39)**
	New-Mar	18.11 (1.02)	4.44 (0.31)	3.74 (0.42)	2.80 (0.58)
4	Lasso	17.57 (1.73)	0.10 (0.31)	7.08 (0.95)	12.90 (2.00)
	AdLasso	17.34 (1.15)	0.16 (0.46)	5.77 (0.55)	10.35 (0.75)
	IVIS	15.28 (0.81)	4.58 (1.65)	6.11 (0.62)	12.78 (0.82)
	New-Lasso	**20.00 (0.00)**	**0.00 (0.00)**	**0.54 (0.06)**	**0.68 (0.04)**
	New-Mar	19.14 (1.18)	9.24 (2.59)	2.38 (0.59)	1.56 (0.22)
5	Lasso	**20.00 (0.00)**	0.10 (0.31)	**0.43 (0.06)**	0.82 (0.09)
	AdLasso	16.74 (1.23)	0.70 (0.64)	6.04 (0.40)	8.36 (0.50)
	IVIS	15.62 (0.88)	3.38 (0.96)	5.93 (0.56)	10.14 (0.63)
	New-Lasso	**20.00 (0.00)**	**0.00 (0.00)**	0.54 (0.07)	**0.70 (0.04)**
	New-Mar	18.30 (1.34)	4.40 (0.74)	2.58 (0.37)	2.04 (0.26)
6	Lasso	15.56 (2.46)	0.24 (0.91)	6.42 (1.04)	11.98 (2.22)
	AdLasso	16.64 (1.19)	0.18 (0.44)	5.21 (0.47)	9.41 (0.74)
	IVIS	14.37 (1.02)	3.16 (1.05)	6.01 (0.69)	10.79 (0.95)
	New-Lasso	**19.65 (0.59)**	**0.00 (0.00)**	**1.16 (0.25)**	**1.08 (0.24)**
	New-Mar	18.14 (1.53)	4.24 (1.77)	3.12 (0.49)	2.46 (0.35)
7	Lasso	14.64 (2.48)	0.16 (0.49)	6.68 (0.92)	12.58 (2.03)
	AdLasso	16.05 (1.43)	0.10 (0.36)	5.33 (0.49)	9.58 (0.65)
	IVIS	15.05 (1.14)	2.94 (0.83)	5.98 (0.68)	11.16 (0.88)
	New-Lasso	**19.77 (0.55)**	**0.00 (0.00)**	**1.02 (0.22)**	**1.00 (0.35)**
	New-Mar	17.65 (1.57)	4.04 (1.88)	3.38 (0.34)	2.72 (0.25)
8	Lasso	16.50 (2.44)	0.50 (0.41)	6.08 (1.17)	11.04 (2.41)
	AdLasso	16.06 (1.46)	0.12 (0.33)	5.38 (0.46)	9.63 (0.69)
	IVIS	14.70 (1.19)	3.32 (1.12)	6.19 (0.73)	11.24 (1.04)
	New-Lasso	**19.50 (0.69)**	**0.00 (0.00)**	**1.36 (0.33)**	**1.24 (0.25)**
	New-Mar	17.63 (1.63)	3.40 (0.30)	3.50 (0.33)	2.84 (0.33)
9	Lasso	15.41 (2.03)	2.60 (1.41)	6.72 (1.10)	5.66 (1.02)
	AdLasso	15.74 (1.57)	0.41 (0.62)	7.62 (0.32)	9.38 (0.52)
	IVIS	14.24 (1.32)	4.63 (1.39)	7.43 (1.07)	11.02 (1.19)
	New-Lasso	**18.30 (1.49)**	**0.00 (0.00)**	**2.52 (0.11)**	**1.56 (0.59)**
	New-Mar	14.45 (2.01)	10.00 (2.97)	5.52 (0.92)	2.58(0.68)

## Data Availability

Publicly available datasets were analyzed in this study. The SKCM and the LUAD datasets can be found here: http://www.cbioportal.org/ (accessed on 16 January 2021), and can be downloaded via the cgdsr R package.

## Appendix A. Estimation under the Accelerated Failure Time Model

The AFT model is an alternative to the commonly used Cox model in survival analysis, and regresses the logarithm of the survival time over the covariates. Consider a sample set $\left\{\left(X^{n},Y^{n}\right):X^{n}\in R^{p},Y^{n}\in R\right\}$ of size *N*, where $X^{n}=\left(X_{1}^{n},\cdots ,X_{p}^{n}\right)$ denotes the *p*-dimensional covariates. Under the right-censoring situation, we obtain $Y^{n}=\min\left\{T^{n},C^{n}\right\}$, where $T^{n}$ and $C^{n}$ denote the survival time and censoring time of the *n*th subject, respectively. Assume *N* subjects have been sorted by a known biomarker. We specify the following AFT model:

$$\log\left(T^{n}\right)=\sum_{j=1}^{p}X_{j}^{n}\beta_{j}^{n}+\varepsilon^{n},\;n=1,2,\cdots ,N,$$

where $\varepsilon^{n}$ is the random error with mean zero.

Unknown coefficients $\beta_{j}={\left(\beta_{j}^{1},\ldots ,\beta_{j}^{N}\right)}^{⊤}$ can be estimated by the weighted least squares method [42], where the weight is defined as a Kaplan-Meier weight. Let $Y^{\left[1\right]}\leq Y^{\left[2\right]}\leq \ldots Y^{\left[n\right]}$ be the order statistics of $Y^{n}$, n=1,2,…,N, and $\delta^{\left[n\right]}$ the associated indicator function. The Kaplan-Meier weight can be computed as:

$$\begin{array}{c} w_{1}=\frac{\delta^{\left[1\right]}}{N}\\ w_{n}=\frac{\delta^{\left[n\right]}}{N-n+1}\prod_{i=1}^{n-1}{\left(\frac{N-i}{N-i+1}\right)}^{\delta^{\left[i\right]}},\;n=2,\ldots ,N. \end{array}$$

The weighted least-square loss function becomes:

$$L\left(\beta \right)=\sum_{n=1}^{N}w_{n}{\left(\log Y^{\left[n\right]}-\sum_{j=1}^{p}X_{j}^{\left[n\right]}\beta_{j}^{\left[n\right]}\right)}^{2},$$

where $X^{\left[n\right]}$ is the vector of the covariates associated with $Y^{\left[n\right]}$ and $\beta_{j}^{\left[n\right]}$’s are the corresponding coefficients.

## Appendix B

**Proof of Theorem 1.** From the definition of $\hat{\beta }$:

(A1) $$\frac{1}{2N}∥\tilde{Y}-\tilde{Z}\hat{\beta }{∥}_{2}^{2}+\lambda_{1}\sum_{j=1}^{p}\frac{1}{∥{\tilde{\beta }}_{j}{∥}_{\infty }}∥{\hat{\beta }}_{j}{∥}_{2}\leq \frac{1}{2N}∥\tilde{Y}-\tilde{Z}\beta^{0}{∥}_{2}^{2}+\lambda_{1}\sum_{j=1}^{p}\frac{1}{∥{\tilde{\beta }}_{j}{∥}_{\infty }}{∥\beta_{j}^{0}∥}_{2}.$$

Let $\tilde{\varepsilon }={\left(\varepsilon^{⊤},{\left(-\sqrt{N\lambda_{2}}A\beta^{0}\right)}^{⊤}\right)}^{⊤}$ with $\varepsilon ={\left(\varepsilon^{1},\ldots ,\varepsilon^{N}\right)}^{⊤}$. From (A1),

(A2) $$\frac{1}{2N}∥\tilde{Z}\beta^{0}-\tilde{Z}\hat{\beta }{∥}_{2}^{2}\leq \lambda_{1}\sum_{j=1}^{p}\omega_{j}(∥\beta_{j}^{0}{∥}_{2}-∥{\hat{\beta }}_{j}{∥}_{2})+\frac{1}{N}{\tilde{\varepsilon }}^{⊤}\tilde{Z}\left(\hat{\beta }-\beta^{0}\right).$$

From the Cauchy-Schwartz inequality, we have:

(A3) $$\begin{array}{cc} \frac{1}{N}{\tilde{\varepsilon }}^{⊤}\tilde{Z}\left(\hat{\beta }-\beta^{0}\right) & \leq \frac{1}{N}\varepsilon^{⊤}Z\left(\hat{\beta }-\beta^{0}\right)+\lambda_{2}∥J\beta^{0}{∥}_{2}{∥\beta^{0}-\hat{\beta }∥}_{2}\\ \; & \leq \frac{1}{N}{m∥\varepsilon ||}_{2}\sum_{j=1}^{p}∥\beta_{j}^{0}-\hat{\beta_{j}}{∥}_{2}+\lambda_{2}∥J\beta^{0}{∥}_{2}{∥\beta^{0}-\hat{\beta }∥}_{2}. \end{array}$$

We define $\kappa_{p}=\max_{1\leq j\leq p}{∥{\tilde{\beta }}_{j}∥}_{\infty }$. Under event Ω, by Lemma 1 in Huang et al. [26], for any 1≤j≤p, we have:

(A4) $$P(\frac{1}{N}{m∥\varepsilon ∥}_{2}>\frac{\lambda_{1}}{∥\tilde{\beta_{j}}{∥}_{\infty }})\leq \exp\left(-{\left[\frac{\kappa_{p}m\log N\sqrt{\log p}}{N^{\frac{3}{4}}\lambda_{1}}\right]}^{2}\right)\to 0.$$

As a result, from (A2):

(A5) $$\begin{array}{cc} \frac{1}{2N}{∥\tilde{Z}\beta^{0}-\tilde{Z}\hat{\beta }∥}_{2}^{2} & \leq \lambda_{1}\sum_{j=1}^{p}\omega_{j}(∥\beta_{j}^{0}{∥}_{2}-∥{\hat{\beta }}_{j}{∥}_{2})+\lambda_{1}\sum_{j=1}^{p}\omega_{j}∥\beta_{j}^{0}-{\hat{\beta }}_{j}{∥}_{2}+\lambda_{2}∥J\beta^{0}{∥}_{2}{∥\beta^{0}-\hat{\beta }∥}_{2}\\ \; & \leq 2\lambda_{1}\sum_{j=1}^{q}\omega_{j}∥\beta_{j}^{0}-{\hat{\beta }}_{j}{∥}_{2}+\lambda_{2}∥J\beta^{0}{∥}_{2}{∥\beta^{0}-\hat{\beta }∥}_{2}\\ \; & \leq 2\lambda_{1}\sqrt{q}\alpha_{2}^{-1}∥\beta^{0}-\hat{\beta }{∥}_{2}+\lambda_{2}∥J\beta^{0}{∥}_{2}{∥\beta^{0}-\hat{\beta }∥}_{2}. \end{array}$$

According to condition (C2), we finally obtain:

(A6) $$∥\beta^{0}-\hat{\beta }{∥}_{2,N}\leq \frac{4\lambda_{1}\sqrt{q}\alpha_{2}^{-1}+2\lambda_{2}{∥J\beta^{0}∥}_{2}}{\tau \sqrt{N}}.$$

This completes the proof of Theorem 1. □

## Appendix C

**Proof of Theorem 2.** Consider the Karush-Kuhn-Tucker (KKT) condition:

(A7) $$-\frac{1}{N}Z_{j}\left(Y-Z\hat{\beta }\right)+\lambda_{2}A^{⊤}A\hat{\beta_{j}}+\lambda_{1}\omega_{j}\frac{\hat{\beta_{j}}}{∥{\hat{\beta }}_{j}{∥}_{2}}=0,\;\;\;\;\;\;\mathrm{if}\;∥{\hat{\beta }}_{j}∥\neq 0$$

(A8) $$-\lambda_{1}\omega_{j}e_{N}\leq \frac{1}{N}Z_{j}\left(Y-Z\hat{\beta }\right)\leq \lambda_{1}\omega_{j}e_{N},\;\;\;\;\;\;\;\;\;\;\mathrm{if}\;∥{\hat{\beta }}_{j}∥=0$$

where $e_{N}$ is a N×1 vector whose elements are all 1s. We define $Z^{*}=\sqrt{N}{\left(\frac{1}{N}Z^{⊤}Z+\lambda_{2}J\right)}^{\frac{1}{2}}$ and $Y^{*}={Z^{*}}^{-1}Z^{⊤}Y$. Therefore, $\hat{\beta }$ is also the minimizer of the following objective function:

(A9) $$\frac{1}{2N}∥Y^{*}-Z^{*}{\beta ||}_{2}^{2}+\lambda_{1}\sum_{j=1}^{p}\omega_{j}{∥\beta_{j}∥}_{2}.$$

As a result, if $∥{\hat{\beta }}_{j}{∥}_{2}\neq 0$ for $j\in E_{1}$, then, by the KKT condition:

(A10) $$-\frac{1}{N}{Z^{*}}_{E_{1}}^{⊤}\left({Y^{*}}_{E_{1}}-{Z^{*}}_{E_{1}}{\hat{\beta }}_{E_{1}}\right)=-W_{E_{1}},$$

where $W_{E_{1}}={\left(W_{1}^{⊤},\cdots ,W_{q}^{⊤}\right)}^{⊤}$ is a N×q vector with $W_{j}=\frac{\lambda_{1}\omega_{j}}{{∥{\hat{\beta }}_{j}∥}_{2}}{\hat{\beta }}_{j}$. Since

(A11) $$\begin{array}{cc} Z^{*}\beta^{0}-E\left(Y^{*}\right) & ={Z^{*}}^{-1}{Z^{*}}^{2}\beta^{0}-{Z^{*}}^{-1}Z^{⊤}Z\beta^{0}\\ \; & ={Z^{*}}^{-1}\left({Z^{*}}^{2}-Z^{⊤}Z\right)\beta^{0}\\ \; & ={Z^{*}}^{-1}\left(N\lambda_{2}J\right)\beta^{0}, \end{array}$$

we have

(A12) $$\begin{array}{cc} {Z^{*}}_{E_{1}}^{⊤}{Y^{*}}_{E_{1}} & ={Z^{*}}_{E_{1}}E\left({Y^{*}}_{E_{1}}\right)+{Z^{*}}_{E_{1}}\left[{Y^{*}}_{E_{1}}-E\left({Y^{*}}_{E_{1}}\right)\right]\\ \; & ={Z^{*}}_{E_{1}}^{2}\beta_{E_{1}}^{0}-N\lambda_{2}J_{E_{1}}\beta_{E_{1}}^{0}+Z_{E_{1}}^{⊤}Y-E\left(Z_{E_{1}}^{⊤}Y\right)\\ \; & =N\Sigma_{E_{1}E_{1}}\beta_{E_{1}}^{0}-N\lambda_{2}J_{E_{1}}\beta_{E_{1}}^{0}+Z_{E_{1}}^{⊤}\varepsilon . \end{array}$$

Let $\hat{u}=\hat{\beta }-\beta^{0}$ and $S=Z^{⊤}\varepsilon /\sqrt{N}$. As a result, if there exists $\hat{u}$ so that:

(A13) $$\Sigma_{E_{1}E_{1}}{\hat{u}}_{E_{1}}-\frac{1}{\sqrt{N}}S_{E_{1}}=-W_{E_{1}}-\lambda_{2}J_{E_{1}}\beta_{E_{1}}^{0}$$

(A14) $$∥{\hat{u}}_{j}{∥}_{2}\leq {∥\beta_{j}^{0}∥}_{2},\;\mathrm{for}\;j\in E_{1}$$

and

(A15) $$∥\frac{1}{N}Z_{j}^{⊤}\left(Y-Z_{E_{1}}{\hat{\beta }}_{E_{1}}\right){∥}_{2}<\sqrt{N}\lambda_{1}\omega_{j},\;\mathrm{for}\;j\in E_{0}.$$

Then, we have $∥{\hat{\beta }}_{j}{∥}_{2}=0\;\mathrm{for}\;j\in E_{0}$, and $∥{\hat{\beta }}_{j}{∥}_{2}\neq 0\;\mathrm{for}\;j\in E_{1}$. From (A3),

(A16) $${\hat{u}}_{E_{1}}-\frac{1}{\sqrt{N}}\Sigma_{E_{1}E_{1}}^{-1}S_{E_{1}}=-\Sigma_{E_{1}E_{1}}^{-1}W_{E_{1}}-\lambda_{2}\Sigma_{E_{1}E_{1}}^{-1}J_{E_{1}}\beta_{E_{1}}^{0}.$$

Then,

(A17) $$\begin{array}{cc} Y-Z_{E_{1}}{\hat{\beta }}_{E_{1}} & =\varepsilon -Z_{E_{1}}\left({\hat{\beta }}_{E_{1}}-\beta_{E_{1}}^{0}\right)\\ \; & =\varepsilon -\frac{1}{N}Z_{E_{1}}\Sigma_{E_{1}E_{1}}^{-1}Z_{E_{1}}^{⊤}\varepsilon +Z_{E_{1}}\Sigma_{E_{1}E_{1}}^{-1}W_{E_{1}}+\lambda_{2}Z_{E_{1}}\Sigma_{E_{1}E_{1}}^{-1}J_{E_{1}}\beta_{E_{1}}^{0}. \end{array}$$

We define $H=I-\frac{1}{N}Z_{E_{1}}\Sigma_{E_{1}E_{1}}^{-1}Z_{E_{1}}^{⊤}$. Then, from (A3)–(A5), if

$$\begin{array}{ccc} & & ∥{\left(\frac{1}{\sqrt{N}}\Sigma_{E_{1}E_{1}}^{-1}S_{E_{1}}-\Sigma_{E_{1}E_{1}}^{-1}\left(W_{E_{1}}+\lambda_{2}J_{E_{1}}\beta_{E_{1}}^{0}\right)\right)}_{j}{∥}_{2}\leq {∥\beta_{j}^{0}∥}_{2},\;\;\forall j\in E_{1}\\ & & ∥\frac{1}{N}Z_{j}^{⊤}\left[H\varepsilon +Z_{E_{1}}\Sigma_{E_{1}E_{1}}^{-1}\left(W_{E_{1}}+\lambda_{2}J_{E_{1}}\beta_{E_{1}}^{0}\right)\right]{∥}_{2}<\sqrt{N}\lambda_{1}\omega_{j},\;\forall j\in E_{0} \end{array}$$

are satisfied, we have $∥{\hat{\beta }}_{j}{∥}_{2}=0\;\mathrm{for}\;j\in E_{0}$ and $∥{\hat{\beta }}_{j}{∥}_{2}\neq 0\;\mathrm{for}\;j\in E_{1}$. We define the events as:

$$D_{1}=\left\{\frac{1}{N}{∥{\left(\Sigma_{E_{1}E_{1}}^{-1}Z_{E_{1}}^{⊤}\varepsilon \right)}_{j}∥}_{2}>\frac{∥\beta_{j}^{0}{∥}_{2}}{2},\exists k\in E_{1}\right\},$$

$$D_{2}=\left\{∥{\left[\Sigma_{E_{1}E_{1}}^{-1}\left(W_{E_{1}}+\lambda_{2}J_{E_{1}}\beta_{E_{1}}^{0}\right)\right]}_{j}{∥}_{2}>\frac{∥\beta_{j}^{0}{∥}_{2}}{2},\exists k\in E_{1}\right\},$$

$$D_{3}=\left\{\frac{1}{\sqrt{N}}{∥Z_{j}H\varepsilon ∥}_{2}>\frac{N\lambda_{1}\omega_{j}}{2},\exists k\in E_{0}\right\},$$

and

$$D_{4}=\left\{\frac{1}{N}{∥Z_{j}Z_{E_{1}}\Sigma_{E_{1}E_{1}}^{-1}\left(W_{E_{1}}+\lambda_{2}J_{E_{1}}\beta_{E_{1}}^{0}\right)∥}_{2}>\frac{\sqrt{N}\lambda_{1}\omega_{j}}{2},\exists k\in E_{0}\right\}.$$

Then, we have:

$$P(∥{\hat{\beta }}_{j}{∥}_{2}\neq 0,j\in E_{0}\;\mathrm{or}\;∥{\hat{\beta }}_{j}{∥}_{2}=0,j\notin E_{0})\leq P\left(D_{1}\right)+P\left(D_{2}\right)+P\left(D_{3}\right)+P\left(D_{4}\right).$$

First, we consider $P(D_{1})$. Because $∥Z_{E_{1}}{∥}_{2}={∥Z_{E_{1}}^{⊤}∥}_{2}=\sup_{X\in R^{N}}\frac{\left|\right|Z_{E_{1}}^{⊤}X{\left|\right|}_{2}}{{\left||X|\right|}_{2}}\leq m\sqrt{q}$, then, for any $j\in E_{1}$,

(A18) $$∥{\left(\Sigma_{E_{1}E_{1}}^{-1}Z_{E_{1}}^{⊤}\varepsilon \right)}_{j}{∥}_{2}\leq ∥\Sigma_{E_{1}E_{1}}^{-1}Z_{E_{1}}^{⊤}{\varepsilon ∥}_{2}\leq ∥\Sigma_{E_{1}E_{1}}^{-1}{∥}_{2}{∥Z_{E_{1}}^{⊤}\varepsilon ∥}_{2}\leq \frac{m\sqrt{q}{∥\varepsilon ∥}_{2}}{\tau }.$$

From condition (C6) and Lemma 1 in Huang et al. [26], we have

(A19) $$P\left(D_{1}\right)\leq P\left(\frac{1}{\sqrt{N}}∥\Sigma_{E_{1}E_{1}}^{-1}Z_{E_{1}}^{⊤}{\varepsilon ∥}_{2}>\frac{N\alpha_{1}}{2}\right)\leq \exp\left(-{\left[\frac{\tau \alpha_{1}N^{\frac{5}{4}}}{2m\sqrt{q}\log N}\right]}^{2}\right)\to 0.$$

For $D_{2}$, we define $R=\{∥{\tilde{\beta }}_{j}{∥}_{\infty }\geq \alpha_{2},j\in E_{1}\}$. Then,

(A20) $$P\left(D_{2}\right)=P\left(D_{2}⋂R\right)+P\left(D_{2}⋂R^{c}\right)\leq P\left(D_{2}⋂R\right)+P\left(R^{c}\right).$$

From condition (C2), $P(R^{c})\to 0$. Then, we only need to prove $P(D_{2}⋂R)\to 0$. Since $\Sigma_{E_{1}E_{1}}^{-1}$ is invertible, we can prove that for any $j\in E_{1}$,

$$\begin{array}{ccc} ∥{\left(\Sigma_{E_{1}E_{1}}^{-1}\left(W_{E_{1}}+\lambda_{2}J_{E_{1}}\beta_{E_{1}}^{0}\right)\right)}_{j}{∥}_{2} & \leq & ∥\Sigma_{E_{1}E_{1}}^{-1}\left(W_{E_{1}}+\lambda_{2}J_{E_{1}}\beta_{E_{1}}^{0}\right){∥}_{2}\\ & \leq & ∥\Sigma_{E_{1}E_{1}}^{-1}W_{E_{1}}{∥}_{2}+∥\Sigma_{E_{1}E_{1}}^{-1}\lambda_{2}J_{E_{1}}\beta_{E_{1}}^{0}{∥}_{2}\\ & \leq & \frac{\lambda_{1}\sqrt{q}\alpha_{2}^{-1}+\lambda_{2}{∥J\beta^{0}∥}_{2}}{\tau }. \end{array}$$

From Condition (C6), we have

(A21) $$\frac{2∥{\left(\Sigma_{E_{1}E_{1}}^{-1}\left(W_{E_{1}}+\lambda_{2}J_{E_{1}}\beta_{E_{1}}^{0}\right)\right)}_{j}{∥}_{2}}{\sqrt{N}\alpha_{1}}\leq \frac{2\lambda_{1}}{\tau \alpha_{1}\alpha_{2}}\sqrt{\frac{q}{N}}+\frac{2\lambda_{2}∥J\beta^{0}∥}{\tau \alpha_{1}\sqrt{N}}\to 0.$$

Therefore, $P(D_{2}⋂R)=0$. Next, we consider $P(D_{3})$. Similarly to above, we define $E=\{||{\tilde{\beta }}_{j}|{|}_{\infty }<\frac{1}{r}+M,j\in E_{0}\}⋂R.$ Then,

$$P\left(D_{3}\right)=P\left(D_{3}⋂E\right)+P\left(D_{3}⋂E^{c}\right)\leq P\left(D_{3}⋂E\right)+P\left(E^{c}\right).$$

Under Conditions (C2) and (C5), we know $P(E^{c})\to 0$ and, thus, only need to prove that $P(D_{3}⋂E)\to 0$. Since $\Sigma_{E_{1}E_{1}}$ is invertible, we have, for any $j\in E_{0}$,

$$∥Z_{j}{H\varepsilon ∥}_{2}\leq {m(∥\varepsilon ∥}_{2}+\frac{m^{2}q{∥\varepsilon ∥}_{2}}{\tau N}).$$

Then,

(A22) $$\begin{array}{cc} P(D_{3}⋂E) & \leq P\left(\frac{2}{\sqrt{N}}{∥Z_{j}H\varepsilon ∥}_{2}>\frac{N\lambda_{1}}{\frac{1}{r}+M},j\in E_{0}\right)\\ & \leq P\left(\frac{1}{\sqrt{N}}{∥\varepsilon ∥}_{2}>\frac{N\lambda_{1}}{2\left(\frac{1}{r}+M\right)\left(m+\frac{m^{3}q}{\tau N}\right)}\right)\\ & \leq \left(p-q\right)q_{N}^{*}\left({\left[\frac{N^{\frac{5}{4}}\lambda_{1}}{2\left(\frac{1}{r}+M\right)\left(m+\frac{m^{3}q}{\tau N}\right)}\right]}^{2}\right), \end{array}$$

where function $q_{N}^{*}\left(\cdot \right)$ is the same as $q_{n}^{*}\left(\cdot \right)$ in Lemma 1 of Huang et al. [26]. Therefore, from Lemma 1 of Huang et al. [26] and Condition (C6), $P(D_{3}⋂E)\to 0$. Finally, we consider $D_{4}$. To prove $P(D_{4})\to 0$, we only need to prove $P(D_{4}⋂E)\to 0$. Since $\Sigma_{E_{1}E_{1}}$ is invertible, we can prove that, for any $j\in E_{0}$,

(A23) $$\begin{array}{c} \frac{1}{N}∥Z_{j}Z_{E_{1}}\Sigma_{E_{1}E_{1}}^{-1}\left(W_{E_{1}}-\lambda_{2}J_{E_{1}}\beta_{E_{1}}^{0}\right){∥}_{2}\leq \frac{m^{2}\sqrt{q}}{\tau N}(\lambda_{1}\alpha_{2}^{-1}\sqrt{q}+\lambda_{2}∥J\beta^{0}{∥}_{2}). \end{array}$$

Under Condition (C6),

(A24) $$\frac{\frac{2}{N}{∥Z_{j}Z_{E_{1}}\Sigma_{E_{1}E_{1}}W_{E_{1}}∥}_{2}}{\sqrt{N}\lambda_{1}\omega_{j}}\leq \frac{2m^{2}\sqrt{q}(\lambda_{1}\alpha_{2}^{-1}\sqrt{q}+\lambda_{2}∥J\beta^{0}{∥}_{2})}{\tau \sqrt{N^{3}}\lambda_{1}}\left(\frac{1}{r}+M\right)\leq 1.$$

Namely, $P(D_{4}⋂E)\to 0$. This completes the proof of Theorem 2. □

**Remark** **A1.**
*We show that the marginal regression estimator satisfies Condition (C5) under some assumptions and can thus be used as the initial estimator. With the standardization of $X=(X_{1},X_{2},\ldots ,X_{p})$, the estimated marginal regression coefficient becomes:*

(A25)
$${\tilde{\beta }}_{k}=\frac{\sum_{n=1}^{N}X_{k}^{n}y^{n}}{\sum_{n=1}^{N}{\left(X_{k}^{n}\right)}^{2}}=\sum_{j=1}^{q}\left(\frac{\sum_{n=1}^{N}X_{k}^{n}X_{j}^{n}\beta_{j}^{0n}}{N}\right)+\frac{X_{k}^{⊤}\varepsilon }{N}.$$

We define

(A26) $$\xi_{k}=\sum_{j=1}^{q}\left(\frac{\sum_{n=1}^{N}X_{k}^{n}X_{j}^{n}\beta_{j}^{0n}}{N}\right).$$

For $k\in E_{1}$, we restrict $\xi_{k}=O\left(N^{\frac{d-1}{2}}\right)$, where $0\leq d\leq \frac{1}{3}$, so that the non-zero coefficients’ signals are bounded away from zero at certain rates.

Similarly to the “partial orthogonality” condition in Huang et al. [26], we assume that the correlation between the covariates with zero coefficients and those with nonzero coefficients (multiplying the corresponding coefficient) is not large, that is,

$$\frac{1}{N}|\sum_{n=1}^{N}X_{k}^{n}\left(X_{j}^{n}\beta_{j}^{0n}\right)|=\frac{1}{N}\left|X_{k}^{⊤}f_{j}\left(X_{j}\right)\right|\leq \rho_{N},\;k\in E_{0},\;j\in E_{1}.$$

For $k\in E_{0}$, we have $|\xi_{k}|\leq q\rho_{N}$. Assume

$$\rho_{N}<\frac{\tau \lambda_{1}\sqrt{N^{3}}}{2m^{2}\sqrt{q^{3}}(\lambda_{1}\alpha_{2}^{-1}\sqrt{q}+\lambda_{2}∥J\beta^{0}{∥}_{2})}.$$

From Condition (C6), $q\rho_{N}<1$. From Lemma 1 in Huang et al. [26], for any ϵ>0, if $r=o\left(\frac{\sqrt{N}}{\log p\log N}\right)$, we have:

(A27) $$P\left(r\max_{1\leq j\leq p}\left|{\tilde{\beta }}_{j}-\xi_{j}\right|>\epsilon \right)=P\left(r\max_{1\leq j\leq p}\frac{X_{j}^{⊤}\varepsilon }{N}>\epsilon \right)\leq pq^{*}\left(\frac{\sqrt{N}\epsilon }{r\log N}\right)=o\left(1\right).$$

When $p=O(e^{N^{\frac{1}{2}-\delta }})$ with 0<δ<0.5, *r* can be set as $O(\frac{N^{\delta -c}}{\log N})$ for a small c>0. Therefore, the marginal regression estimator satisfies Condition (C5).

## Appendix D. More Tables and Figures

**Table A1:** Simulation results for *N* = 200, *p* = 500, *q* = 20, and ρ=0.8. Each cell shows the mean (sd). The bold represents the best value.

Scenario	Method	TP	FP	RMSE	RPE
1	Lasso	13.61 (2.37)	0.20 (0.41)	5.87 (0.50)	14.08 (2.48)
	AdLasso	16.23 (1.54)	0.26 (0.83)	5.05 (0.46)	10.10 (0.39)
	IVIS	12.85 (1.43)	2.88 (1.37)	5.98 (1.02)	11.72 (0.92)
	New-Lasso	15.56 (1.39)	**0.00 (0.00)**	4.48 (0.82)	2.66 (0.54)
	New-Mar	**20.00 (0.00)**	0.56 (0.19)	**1.12 (0.17)**	**0.82 (0.04)**
2	Lasso	11.50 (1.67)	0.41 (0.82)	6.72 (0.45)	18.20 (1.97)
	AdLasso	16.90 (1.06)	**0.11 (0.31)**	5.20 (0.44)	10.38 (0.44)
	IVIS	12.89 (1.13)	3.03 (0.86)	6.04 (0.78)	11.95 (0.96)
	New-Lasso	15.37 (1.53)	0.16 (0.07)	4.90 (0.95)	2.94 (0.64)
	New-Mar	**20.00 (0.00)**	0.70 (0.16)	**0.84 (0.10)**	**0.72 (0.05)**
3	Lasso	12.90 (2.9)	0.10 (0.31)	7.32 (0.80)	18.60 (3.54)
	AdLasso	16.80 (1.32)	0.07 (0.25)	5.55 (0.69)	11.16 (0.54)
	IVIS	13.33 (0.96)	2.92 (1.01)	6.55 (1.15)	12.65 (1.24)
	New-Lasso	15.61 (1.73)	**0.04 (0.02)**	5.56 (1.17)	3.10 (0.69)
	New-Mar	**20.00 (0.00)**	0.56 (0.15)	**0.96 (0.14)**	**0.76 (0.05)**
4	Lasso	14.03 (2.27)	0.20 (0.05)	7.56 (0.82)	18.54 (3.21)
	AdLasso	17.34 (1.49)	0.13 (0.51)	6.02 (0.85)	12.29 (0.48)
	IVIS	14.35 (0.93)	3.75 (0.92)	6.47 (0.73)	12.43 (0.97)
	New-Lasso	16.90 (1.45)	**0.08 (0.01)**	5.26 (1.39)	2.86 (0.80)
	New-Mar	**20.00 (0.00)**	0.84 (0.75)	**0.92 (0.10)**	**0.70 (0.04)**
5	Lasso	**20.00 (0.00)**	1.02 (0.27)	**0.50 (0.10)**	**0.69 (0.07)**
	AdLasso	17.08 (1.37)	0.07 (0.24)	5.36 (0.67)	11.13 (0.49)
	IVIS	13.54 (0.81)	3.24 (0.90)	6.07 (0.69)	11.06 (0.7)
	New-Lasso	19.87 (0.37)	**0.00 (0.00)**	1.02 (0.49)	0.79 (0.14)
	New-Mar	**20.00 (0.00)**	0.84 (0.23)	0.90 (0.12)	0.72 (0.03)
6	Lasso	15.23 (2.65)	0.33 (0.80)	6.18 (0.78)	13.52 (2.50)
	AdLasso	17.09 (1.14)	0.06 (0.24)	5.39 (0.49)	11.03 (0.44)
	IVIS	13.40 (1.05)	3.04 (0.97)	6.05 (0.89)	12.21 (0.9)
	New-Lasso	17.00 (1.75)	**0.00 (0.00)**	4.36 (1.43)	2.02 (0.68)
	New-Mar	**20.00 (0.00)**	1.44 (0.33)	**1.16 (0.14)**	**0.74 (0.05)**
7	Lasso	16.25 (2.29)	0.37 (0.81)	5.90 (0.77)	11.86 (2.23)
	AdLasso	17.28 (1.11)	0.13 (0.46)	5.30 (0.61)	10.99 (0.41)
	IVIS	13.76 (0.99)	2.82 (1.15)	5.97 (0.86)	12.22 (0.92)
	New-Lasso	16.90 (1.07)	**0.00 (0.00)**	4.38 (0.85)	2.02 (0.38)
	New-Mar	**19.95 (0.22)**	1.10 (0.21)	**1.22 (0.40)**	**0.80 (0.14)**
8	Lasso	16.15 (2.18)	0.16 (0.37)	5.80 (0.93)	11.80 (2.11)
	AdLasso	16.75 (1.79)	0.10 (0.36)	6.08 (0.46)	10.08 (0.39)
	IVIS	13.03 (1.22)	3.20 (1.21)	6.11 (0.92)	12.39 (1.1)
	New-Lasso	16.70 (2.03)	**0.00 (0.00)**	4.50 (1.63)	2.06 (0.78)
	New-Mar	**19.90 (0.31)**	0.96 (0.15)	**1.36 (0.59)**	**0.84 (0.25)**

**Table A2:** Simulation results for *N* = 500, *p* = 500, *q* = 40, and ρ=0.3. Each cell shows the mean (sd). The bold represents the best value.

Scenario	Method	TP	FP	RMSE	RPE
1	Lasso	36.85 (0.37)	0.60 (0.67)	9.20 (0.45)	10.78 (0.74)
	AdLasso	36.00 (1.58)	0.14 (0.45)	10.21 (0.61)	12.01 (0.76)
	IVIS	32.92 (1.63)	6.51 (1.77)	12.66 (1.32)	14.36 (1.05)
	New-Lasso	**39.71 (0.66)**	**0.08 (0.10)**	**1.78 (0.24)**	**0.86 (0.14)**
	New-Mar	35.64 (2.60)	1.20 (0.28)	6.32 (1.12)	3.46 (0.56)
2	Lasso	35.70 (1.69)	**0.92 (0.83)**	8.53 (0.48)	10.14 (0.42)
	AdLasso	35.91 (2.16)	0.84 (1.02)	8.58 (0.57)	9.48 (0.64)
	IVIS	33.56 (1.04)	6.94 (0.95)	11.25 (1.23)	14.74 (0.95)
	New-Lasso	**38.04 (1.50)**	**0.63 (0.10)**	**3.76 (0.45)**	**1.38 (0.46)**
	New-Mar	35.83 (2.09)	1.83 (0.29)	5.92 (0.36)	2.24 (0.38)
3	Lasso	34.47 (2.26)	**0.00 (0.00)**	17.78 (1.89)	21.82 (2.38)
	AdLasso	36.64 (1.38)	0.04 (0.20)	10.18 (0.62)	7.62 (0.47)
	IVIS	33.08 (1.03)	8.25 (2.34)	12.07 (1.84)	16.28 (1.12)
	New-Lasso	**40.00 (0.00)**	**0.00 (0.00)**	**1.26 (0.08)**	**0.60 (0.02)**
	New-Mar	36.51 (0.83)	3.40 (0.95)	4.62 (1.10)	2.20 (0.74)
4	Lasso	31.35 (3.91)	0.20 (0.52)	30.34 (2.06)	37.18 (2.82)
	AdLasso	36.24 (1.41)	**0.10 (0.31)**	12.03 (0.73)	17.19 (0.83)
	IVIS	34.27 (2.45)	9.76 (2.22)	14.49 (2.31)	19.24 (2.69)
	New-Lasso	**39.96 (0.22)**	0.69 (0.10)	**1.42 (0.27)**	**0.68 (0.18)**
	New-Mar	37.40 (0.68)	5.60 (0.54)	6.48 (0.31)	1.16 (0.33)
5	Lasso	**40.00 (0.00)**	0.70 (0.88)	**0.79 (0.10)**	1.30 (0.09)
	AdLasso	35.67 (1.75)	0.12 (0.39)	13.88 (0.59)	12.16 (0.75)
	IVIS	34.97 (1.2)	5.29 (1.65)	14.69 (1.44)	16.20 (1.26)
	New-Lasso	**40.00 (0.00)**	**0.00 (0.00)**	1.04 (0.06)	**0.58 (0.03)**
	New-Mar	37.54 (1.90)	2.41 (0.98)	5.46 (1.50)	2.16 (0.16)
6	Lasso	34.64 (1.35)	0.74 (0.90)	12.08 (0.70)	13.94 (1.11)
	AdLasso	35.58 (0.88)	**0.08 (0.27)**	7.78 (0.50)	8.43 (0.51)
	IVIS	33.80 (1.59)	7.66 (2.80)	11.47 (1.70)	15.37 (1.06)
	New-Lasso	**37.54 (1.39)**	0.10 (0.27)	**5.16 (0.84)**	**2.92 (0.44)**
	New-Mar	33.89 (2.19)	6.10 (2.78)	9.66 (2.17)	3.34 (0.77)
7	Lasso	34.70 (2.13)	0.91 (1.02)	13.26 (0.82)	15.32 (1.36)
	AdLasso	35.64 (0.56)	**0.08 (0.27)**	7.79 (0.48)	8.31 (0.46)
	IVIS	33.09 (1.7)	7.72 (2.13)	11.28 (1.90)	14.02 (1.18)
	New-Lasso	**37.36 (1.73)**	0.12 (0.31)	**5.72 (0.50)**	**2.64 (0.31)**
	New-Mar	33.40 (2.11)	6.32 (2.86)	10.02 (1.03)	3.56 (0.44)
8	Lasso	34.30 (2.18)	0.88 (0.73)	13.20 (0.73)	14.38 (1.18)
	AdLasso	35.45 (0.73)	0.20 (0.40)	7.72 (0.54)	8.25 (0.48)
	IVIS	34.38 (1.79)	7.04 (1.09)	11.43 (1.70)	14.37 (2.21)
	New-Lasso	**37.71 (1.54)**	**0.00 (0.00)**	**5.22 (0.62)**	**3.04 (0.41)**
	New-Mar	32.03 (2.35)	5.84 (2.41)	9.88 (1.76)	3.74 (0.99)
9	Lasso	28.83 (3.54)	0.40 (0.68)	15.42 (1.68)	14.12 (1.12)
	AdLasso	35.34 (1.58)	0.20 (0.48)	21.36 (0.52)	12.02 (0.75)
	IVIS	33.11 (1.20)	6.42 (1.54)	16.94 (2.01)	15.97 (1.95)
	New-Lasso	**37.56 (1.19)**	**0.94 (0.29)**	**4.46 (1.10)**	**1.31 (0.28)**
	New-Mar	24.82 (2.88)	9.55 (0.80)	12.50 (1.56)	3.88 (0.73)

**Table A3:** Simulation results for *N* = 200, *p* = 500, *q* = 40, and ρ=0.8. Each cell shows the mean (sd). The bold represents the best value.

Scenario	Method	TP	FP	RMSE	RPE
1	Lasso	31.56 (2.17)	0.36 (0.33)	13.40 (0.67)	18.82 (1.86)
	AdLasso	32.52 (1.54)	0.14 (0.40)	9.66 (0.75)	13.28 (0.49)
	IVIS	29.46 (0.87)	6.32 (1.21)	13.22 (0.76)	20.86 (0.85)
	New-Lasso	32.96 (2.21)	**0.00 (0.00)**	11.4 (1.40)	3.58 (0.61)
	New-Mar	**39.84 (0.37)**	0.44 (0.09)	**2.16 (0.40)**	**0.70 (0.10)**
2	Lasso	30.60 (2.44)	0.07 (0.31)	11.76 (0.45)	20.90 (2.16)
	AdLasso	31.06 (2.20)	0.02 (0.14)	8.60 (0.69)	10.08 (0.53)
	IVIS	30.46 (1.68)	5.31 (0.91)	13.22 (0.85)	21.86 (0.98)
	New-Lasso	31.80 (1.94)	**0.00 (0.00)**	11.76 (1.14)	4.18 (0.65)
	New-Mar	**39.60 (0.68)**	0.23 (0.15)	**2.58 (0.47)**	**0.78 (0.18)**
3	Lasso	34.30 (2.88)	0.10 (0.30)	19.28 (1.83)	24.90 (2.63)
	AdLasso	32.04 (1.40)	**0.00 (0.00)**	13.20 (1.43)	17.40 (0.81)
	IVIS	31.86 (1.49)	7.37 (1.36)	14.38 (1.28)	25.43 (1.3)
	New-Lasso	33.20 (2.17)	0.03 (0.02)	12.92 (3.07)	3.76 (0.77)
	New-Mar	**40.00 (0.00)**	0.60 (0.08)	**2.12 (0.17)**	**0.66 (0.03)**
4	Lasso	32.85 (3.45)	0.20 (0.41)	31.06 (1.42)	38.52 (3.74)
	AdLasso	33.34 (0.82)	**0.15 (0.55)**	16.36 (1.55)	18.56 (0.83)
	IVIS	31.15 (1.57)	8.13 (1.44)	19.42 (1.93)	28.65 (2.23)
	New-Lasso	32.90 (2.47)	0.96 (0.12)	23.28 (3.47)	5.54 (1.04)
	New-Mar	**40.00 (0.00)**	0.90 (0.19)	**1.76 (0.13)**	**0.60 (0.03)**
5	Lasso	**40.00 (0.00)**	1.44 (0.74)	**0.69 (0.08)**	1.01 (0.03)
	AdLasso	33.53 (1.54)	0.18 (0.44)	12.34 (0.76)	13.28 (0.49)
	IVIS	30.48 (1.38)	6.44 (0.93)	16.01 (2.53)	21.08 (1.54)
	New-Lasso	**40.00 (0.00)**	**0.00 (0.00)**	1.66 (0.13)	0.64 (0.06)
	New-Mar	**40.00 (0.00)**	0.84 (0.10)	1.72 (0.15)	**0.62 (0.02)**
6	Lasso	30.81 (2.35)	0.22 (0.57)	16.80 (1.59)	22.90 (2.74)
	AdLasso	33.82 (2.08)	0.08 (0.34)	13.78 (1.24)	19.28 (0.81)
	IVIS	30.42 (2.02)	6.24 (0.97)	15.49 (1.86)	21.18 (1.14)
	New-Lasso	32.40 (2.09)	**0.00 (0.00)**	11.06 (2.49)	3.18 (0.90)
	New-Mar	**39.80 (0.41)**	1.76 (0.27)	**2.48 (1.22)**	**0.72 (0.21)**
7	Lasso	31.40 (2.35)	0.31 (0.32)	18.38 (1.02)	23.54 (2.13)
	AdLasso	34.45 (1.81)	0.10 (0.42)	13.32 (1.21)	18.16 (0.65)
	IVIS	31.65 (2.31)	5.43 (1.24)	15.13 (1.35)	22.11 (1.47)
	New-Lasso	32.71 (1.69)	**0.04 (0.02)**	12.90 (2.15)	3.62 (0.66)
	New-Mar	**39.65 (0.67)**	1.57 (0.39)	**3.06 (1.03)**	**0.88 (0.16)**
8	Lasso	30.50 (1.99)	0.40 (0.41)	17.48 (1.18)	22.74 (2.19)
	AdLasso	32.04 (1.86)	0.12 (0.39)	13.04 (1.12)	17.84 (0.79)
	IVIS	30.96 (2.58)	6.68 (1.75)	15.22 (2.55)	23.64 (3.41)
	New-Lasso	32.23 (1.80)	**0.00 (0.00)**	12.70 (2.05)	3.64 (0.80)
	New-Mar	**39.51 (0.61)**	1.85 (0.29)	**3.48 (0.68)**	**0.96 (0.22)**

**Table A4:** Data analysis: comparison of variable selection results. Each cell shows the number of overlapping identifications.

	New	Lasso	AdLasso	IVIS
SKCM Dat				
New	6	6	4	3
Lasso		38	12	6
AdLasso			25	5
IVIS				21
LUAD data				
New	7	3	4	3
Lasso		29	8	5
AdLasso			27	4
IVIS				25

**Table A5:** Simulation results for SKCM dataset. Each cell shows the mean (sd). The bold represents the best value.

Method	TP	FP	RMSE	RPE
Lasso	1.70 (1.09)	6.60 (2.12)	1.37 (0.05)	1.30 (0.04)
AdLasso	2.60 (0.70)	4.40 (2.37)	1.35 (0.09)	1.18 (0.11)
IVIS	1.88 (0.69)	11.47 (2.30)	1.66 (0.07)	1.26 (0.13)
New-Lasso	**3.43 (0.53)**	**3.25 (2.43)**	**1.22 (0.11)**	**0.95 (0.05)**
New-Mar	2.96 (0.89)	8.20 (2.09)	1.36 (0.15)	1.04 (0.06)
